# Supplementary material for: Modeling glioblastoma heterogeneity as a dynamic network of cell states
Source: Mol Syst Biol. 2021 Sep 16;17(9):e10105. doi: 10.15252/msb.202010105 (PMC8444284; doi:10.15252/msb.202010105)
Supplement: Supplementary file 6 — Source Data for Figure 5 [file MSB-17-e10105-s004.zip › Figure5A_sourcedata/GSEA_3071/nosigdb_stateII.GseaPreranked.1622795151171/RICHARDS_NOWAKOWSKI_SCIENCE_2017_MGE_IPC2_UPREG.html]

Details for gene set RICHARDS\_NOWAKOWSKI\_SCIENCE\_2017\_MGE\_IPC2\_UPREG[GSEA]

|  || Dataset | state23071 |
| Phenotype | NoPhenotypeAvailable |
| Upregulated in class | na\_pos |
| GeneSet | RICHARDS\_NOWAKOWSKI\_SCIENCE\_2017\_MGE\_IPC2\_UPREG |
| Enrichment Score (ES) | 0.5609196 |
| Normalized Enrichment Score (NES) | 4.2383175 |
| Nominal p-value | 0.0 |
| FDR q-value | 0.0 |
| FWER p-Value | 0.0 |
Table: GSEA Results Summary

  

Fig 1: Enrichment plot: RICHARDS\_NOWAKOWSKI\_SCIENCE\_2017\_MGE\_IPC2\_UPREG      
 Profile of the Running ES Score & Positions of GeneSet Members on the Rank Ordered List

  

| PROBE | GENE SYMBOL | GENE\_TITLE | RANK IN GENE LIST | RANK METRIC SCORE | RUNNING ES | CORE ENRICHMENT || 1 | HIST1H1D |  |  | 0 | 2.323 | 0.0077 | Yes |
| 2 | CLSPN |  |  | 1 | 1.984 | 0.0143 | Yes |
| 3 | HIST1H4C |  |  | 2 | 1.902 | 0.0207 | Yes |
| 4 | RRM2 |  |  | 3 | 1.857 | 0.0268 | Yes |
| 5 | ANLN |  |  | 4 | 1.760 | 0.0327 | Yes |
| 6 | HIST1H1B |  |  | 7 | 1.669 | 0.0377 | Yes |
| 7 | TYMS |  |  | 8 | 1.665 | 0.0432 | Yes |
| 8 | MCM10 |  |  | 9 | 1.654 | 0.0487 | Yes |
| 9 | CDK1 |  |  | 14 | 1.540 | 0.0528 | Yes |
| 10 | DIAPH3 |  |  | 15 | 1.531 | 0.0579 | Yes |
| 11 | ZWINT |  |  | 16 | 1.510 | 0.0629 | Yes |
| 12 | HJURP |  |  | 17 | 1.490 | 0.0679 | Yes |
| 13 | ATAD5 |  |  | 18 | 1.469 | 0.0727 | Yes |
| 14 | FEN1 |  |  | 19 | 1.464 | 0.0776 | Yes |
| 15 | BRCA2 |  |  | 22 | 1.445 | 0.0819 | Yes |
| 16 | UBE2C |  |  | 23 | 1.438 | 0.0867 | Yes |
| 17 | CENPU |  |  | 24 | 1.416 | 0.0914 | Yes |
| 18 | RAD51AP1 |  |  | 25 | 1.384 | 0.0960 | Yes |
| 19 | CENPK |  |  | 26 | 1.382 | 0.1006 | Yes |
| 20 | MYBL2 |  |  | 28 | 1.362 | 0.1048 | Yes |
| 21 | FAM111B |  |  | 29 | 1.361 | 0.1094 | Yes |
| 22 | FBXO5 |  |  | 30 | 1.359 | 0.1139 | Yes |
| 23 | USP1 |  |  | 31 | 1.357 | 0.1184 | Yes |
| 24 | DTL |  |  | 33 | 1.330 | 0.1226 | Yes |
| 25 | RRM1 |  |  | 37 | 1.310 | 0.1261 | Yes |
| 26 | ORC6 |  |  | 39 | 1.308 | 0.1302 | Yes |
| 27 | GINS2 |  |  | 40 | 1.306 | 0.1345 | Yes |
| 28 | CDCA5 |  |  | 41 | 1.289 | 0.1388 | Yes |
| 29 | TK1 |  |  | 42 | 1.281 | 0.1431 | Yes |
| 30 | AURKB |  |  | 44 | 1.276 | 0.1471 | Yes |
| 31 | MCM7 |  |  | 45 | 1.273 | 0.1513 | Yes |
| 32 | HELLS |  |  | 46 | 1.259 | 0.1555 | Yes |
| 33 | MCM8 |  |  | 48 | 1.253 | 0.1594 | Yes |
| 34 | BRCA1 |  |  | 49 | 1.249 | 0.1635 | Yes |
| 35 | CCNA2 |  |  | 53 | 1.230 | 0.1668 | Yes |
| 36 | TICRR |  |  | 55 | 1.227 | 0.1706 | Yes |
| 37 | SKA3 |  |  | 57 | 1.215 | 0.1744 | Yes |
| 38 | PCNA |  |  | 58 | 1.214 | 0.1785 | Yes |
| 39 | HIST1H1C |  |  | 60 | 1.206 | 0.1822 | Yes |
| 40 | MAD2L1 |  |  | 61 | 1.199 | 0.1862 | Yes |
| 41 | UBE2T |  |  | 62 | 1.192 | 0.1902 | Yes |
| 42 | ATAD2 |  |  | 63 | 1.189 | 0.1941 | Yes |
| 43 | PRIM1 |  |  | 65 | 1.170 | 0.1977 | Yes |
| 44 | MSH2 |  |  | 66 | 1.163 | 0.2016 | Yes |
| 45 | RFC3 |  |  | 67 | 1.157 | 0.2055 | Yes |
| 46 | SPC25 |  |  | 68 | 1.156 | 0.2093 | Yes |
| 47 | MELK |  |  | 69 | 1.145 | 0.2131 | Yes |
| 48 | POLD3 |  |  | 70 | 1.143 | 0.2169 | Yes |
| 49 | WDHD1 |  |  | 71 | 1.133 | 0.2207 | Yes |
| 50 | TOP2A |  |  | 72 | 1.132 | 0.2244 | Yes |
| 51 | KIF2C |  |  | 73 | 1.132 | 0.2282 | Yes |
| 52 | MCM5 |  |  | 74 | 1.128 | 0.2320 | Yes |
| 53 | DSN1 |  |  | 75 | 1.122 | 0.2357 | Yes |
| 54 | CCNE2 |  |  | 76 | 1.121 | 0.2394 | Yes |
| 55 | IDH2 |  |  | 78 | 1.117 | 0.2429 | Yes |
| 56 | UHRF1 |  |  | 79 | 1.113 | 0.2466 | Yes |
| 57 | FAM111A |  |  | 80 | 1.112 | 0.2503 | Yes |
| 58 | MCM4 |  |  | 81 | 1.110 | 0.2540 | Yes |
| 59 | RBL1 |  |  | 82 | 1.110 | 0.2577 | Yes |
| 60 | GMNN |  |  | 83 | 1.108 | 0.2613 | Yes |
| 61 | RBBP8 |  |  | 86 | 1.100 | 0.2645 | Yes |
| 62 | KIFC1 |  |  | 88 | 1.094 | 0.2678 | Yes |
| 63 | HIST1H1E |  |  | 90 | 1.088 | 0.2712 | Yes |
| 64 | SKA1 |  |  | 91 | 1.078 | 0.2748 | Yes |
| 65 | XRCC2 |  |  | 92 | 1.076 | 0.2784 | Yes |
| 66 | VRK1 |  |  | 93 | 1.074 | 0.2819 | Yes |
| 67 | NDC80 |  |  | 94 | 1.074 | 0.2855 | Yes |
| 68 | MASTL |  |  | 96 | 1.063 | 0.2888 | Yes |
| 69 | TMPO |  |  | 99 | 1.056 | 0.2917 | Yes |
| 70 | NCAPG |  |  | 100 | 1.056 | 0.2953 | Yes |
| 71 | DHFR |  |  | 101 | 1.051 | 0.2988 | Yes |
| 72 | MYBL1 |  |  | 102 | 1.043 | 0.3022 | Yes |
| 73 | MND1 |  |  | 104 | 1.036 | 0.3054 | Yes |
| 74 | MCM3 |  |  | 105 | 1.035 | 0.3088 | Yes |
| 75 | MKI67 |  |  | 107 | 1.031 | 0.3120 | Yes |
| 76 | STIL |  |  | 108 | 1.031 | 0.3154 | Yes |
| 77 | BRIP1 |  |  | 110 | 1.029 | 0.3186 | Yes |
| 78 | TPX2 |  |  | 111 | 1.025 | 0.3220 | Yes |
| 79 | RFC2 |  |  | 112 | 1.022 | 0.3254 | Yes |
| 80 | CDCA8 |  |  | 113 | 1.018 | 0.3288 | Yes |
| 81 | KIF11 |  |  | 115 | 1.014 | 0.3319 | Yes |
| 82 | NCAPH |  |  | 116 | 1.013 | 0.3353 | Yes |
| 83 | NCAPD3 |  |  | 117 | 1.013 | 0.3386 | Yes |
| 84 | DUT |  |  | 118 | 1.009 | 0.3420 | Yes |
| 85 | SMC2 |  |  | 123 | 1.005 | 0.3443 | Yes |
| 86 | NUF2 |  |  | 125 | 1.004 | 0.3473 | Yes |
| 87 | DEPDC1 |  |  | 126 | 0.999 | 0.3506 | Yes |
| 88 | CHAF1A |  |  | 127 | 0.999 | 0.3540 | Yes |
| 89 | CKS1B |  |  | 128 | 0.999 | 0.3573 | Yes |
| 90 | CENPL |  |  | 132 | 0.989 | 0.3598 | Yes |
| 91 | KIF20B |  |  | 133 | 0.989 | 0.3631 | Yes |
| 92 | MGME1 |  |  | 135 | 0.988 | 0.3661 | Yes |
| 93 | FANCI |  |  | 138 | 0.984 | 0.3688 | Yes |
| 94 | WDR76 |  |  | 142 | 0.975 | 0.3713 | Yes |
| 95 | ESCO2 |  |  | 144 | 0.971 | 0.3742 | Yes |
| 96 | EXO1 |  |  | 145 | 0.969 | 0.3774 | Yes |
| 97 | CKAP2L |  |  | 148 | 0.959 | 0.3801 | Yes |
| 98 | FANCB |  |  | 151 | 0.956 | 0.3827 | Yes |
| 99 | BLM |  |  | 156 | 0.942 | 0.3848 | Yes |
| 100 | NCAPG2 |  |  | 159 | 0.937 | 0.3874 | Yes |
| 101 | KIF23 |  |  | 161 | 0.936 | 0.3902 | Yes |
| 102 | RTKN2 |  |  | 166 | 0.931 | 0.3922 | Yes |
| 103 | GTSE1 |  |  | 167 | 0.929 | 0.3953 | Yes |
| 104 | CDCA2 |  |  | 171 | 0.922 | 0.3976 | Yes |
| 105 | MCM6 |  |  | 176 | 0.906 | 0.3995 | Yes |
| 106 | CENPM |  |  | 178 | 0.901 | 0.4022 | Yes |
| 107 | E2F7 |  |  | 179 | 0.901 | 0.4052 | Yes |
| 108 | HIST1H3D |  |  | 195 | 0.876 | 0.4041 | Yes |
| 109 | C21orf58 |  |  | 201 | 0.873 | 0.4057 | Yes |
| 110 | PLK4 |  |  | 205 | 0.870 | 0.4077 | Yes |
| 111 | NCAPH2 |  |  | 207 | 0.868 | 0.4104 | Yes |
| 112 | PRIM2 |  |  | 212 | 0.865 | 0.4122 | Yes |
| 113 | H2AFZ |  |  | 213 | 0.864 | 0.4150 | Yes |
| 114 | PBK |  |  | 214 | 0.864 | 0.4179 | Yes |
| 115 | CEP152 |  |  | 217 | 0.860 | 0.4202 | Yes |
| 116 | CDCA3 |  |  | 219 | 0.858 | 0.4228 | Yes |
| 117 | BUB1B |  |  | 223 | 0.851 | 0.4248 | Yes |
| 118 | CCDC15 |  |  | 230 | 0.845 | 0.4260 | Yes |
| 119 | ASPM |  |  | 231 | 0.842 | 0.4288 | Yes |
| 120 | EZH2 |  |  | 233 | 0.839 | 0.4314 | Yes |
| 121 | MIS18A |  |  | 239 | 0.833 | 0.4328 | Yes |
| 122 | DBF4 |  |  | 240 | 0.832 | 0.4355 | Yes |
| 123 | RACGAP1 |  |  | 241 | 0.831 | 0.4383 | Yes |
| 124 | PRC1 |  |  | 242 | 0.830 | 0.4411 | Yes |
| 125 | TUBB6 |  |  | 244 | 0.828 | 0.4436 | Yes |
| 126 | KNTC1 |  |  | 249 | 0.825 | 0.4452 | Yes |
| 127 | LMNB1 |  |  | 251 | 0.824 | 0.4477 | Yes |
| 128 | CHEK1 |  |  | 252 | 0.822 | 0.4504 | Yes |
| 129 | KIF18B |  |  | 254 | 0.815 | 0.4529 | Yes |
| 130 | NUP155 |  |  | 257 | 0.810 | 0.4550 | Yes |
| 131 | DNA2 |  |  | 263 | 0.797 | 0.4563 | Yes |
| 132 | DNMT1 |  |  | 265 | 0.792 | 0.4587 | Yes |
| 133 | CENPI |  |  | 269 | 0.788 | 0.4605 | Yes |
| 134 | TUBB4B |  |  | 271 | 0.784 | 0.4628 | Yes |
| 135 | HMGB2 |  |  | 276 | 0.780 | 0.4644 | Yes |
| 136 | SMC4 |  |  | 279 | 0.777 | 0.4664 | Yes |
| 137 | CENPE |  |  | 284 | 0.775 | 0.4679 | Yes |
| 138 | RFWD3 |  |  | 290 | 0.769 | 0.4691 | Yes |
| 139 | BARD1 |  |  | 295 | 0.760 | 0.4706 | Yes |
| 140 | DEK |  |  | 302 | 0.754 | 0.4715 | Yes |
| 141 | MMS22L |  |  | 306 | 0.747 | 0.4731 | Yes |
| 142 | CENPO |  |  | 307 | 0.747 | 0.4756 | Yes |
| 143 | KIF4A |  |  | 309 | 0.746 | 0.4778 | Yes |
| 144 | CKS2 |  |  | 312 | 0.745 | 0.4798 | Yes |
| 145 | PRR11 |  |  | 314 | 0.743 | 0.4820 | Yes |
| 146 | ALYREF |  |  | 315 | 0.741 | 0.4844 | Yes |
| 147 | CENPN |  |  | 320 | 0.737 | 0.4858 | Yes |
| 148 | CCNF |  |  | 321 | 0.737 | 0.4883 | Yes |
| 149 | ANP32B |  |  | 325 | 0.734 | 0.4899 | Yes |
| 150 | IGF2BP1 |  |  | 327 | 0.731 | 0.4921 | Yes |
| 151 | CENPW |  |  | 330 | 0.727 | 0.4939 | Yes |
| 152 | SPC24 |  |  | 340 | 0.719 | 0.4939 | Yes |
| 153 | LIG1 |  |  | 341 | 0.718 | 0.4963 | Yes |
| 154 | ARHGAP11A |  |  | 346 | 0.712 | 0.4976 | Yes |
| 155 | DTYMK |  |  | 364 | 0.701 | 0.4953 | Yes |
| 156 | TTK |  |  | 365 | 0.701 | 0.4977 | Yes |
| 157 | POLE2 |  |  | 367 | 0.700 | 0.4997 | Yes |
| 158 | CENPJ |  |  | 376 | 0.695 | 0.4999 | Yes |
| 159 | TMEM106C |  |  | 377 | 0.694 | 0.5022 | Yes |
| 160 | KIF14 |  |  | 378 | 0.693 | 0.5045 | Yes |
| 161 | E2F8 |  |  | 379 | 0.691 | 0.5068 | Yes |
| 162 | PARPBP |  |  | 382 | 0.688 | 0.5085 | Yes |
| 163 | RFC4 |  |  | 385 | 0.686 | 0.5103 | Yes |
| 164 | KIF18A |  |  | 392 | 0.683 | 0.5109 | Yes |
| 165 | EXOSC9 |  |  | 399 | 0.679 | 0.5116 | Yes |
| 166 | DIAPH1 |  |  | 400 | 0.678 | 0.5138 | Yes |
| 167 | KIF15 |  |  | 404 | 0.676 | 0.5153 | Yes |
| 168 | SMCHD1 |  |  | 406 | 0.676 | 0.5173 | Yes |
| 169 | ZNF367 |  |  | 408 | 0.673 | 0.5192 | Yes |
| 170 | TTF2 |  |  | 413 | 0.671 | 0.5204 | Yes |
| 171 | SAE1 |  |  | 418 | 0.669 | 0.5215 | Yes |
| 172 | KPNA2 |  |  | 420 | 0.669 | 0.5235 | Yes |
| 173 | FOXM1 |  |  | 423 | 0.667 | 0.5252 | Yes |
| 174 | SMC3 |  |  | 425 | 0.667 | 0.5271 | Yes |
| 175 | RMI1 |  |  | 433 | 0.660 | 0.5274 | Yes |
| 176 | BUB3 |  |  | 441 | 0.657 | 0.5277 | Yes |
| 177 | POLA1 |  |  | 443 | 0.654 | 0.5296 | Yes |
| 178 | NCAPD2 |  |  | 457 | 0.647 | 0.5283 | Yes |
| 179 | RAD18 |  |  | 465 | 0.642 | 0.5285 | Yes |
| 180 | POLH |  |  | 487 | 0.635 | 0.5250 | Yes |
| 181 | BIRC5 |  |  | 505 | 0.628 | 0.5225 | Yes |
| 182 | SEPT10 |  |  | 507 | 0.627 | 0.5243 | Yes |
| 183 | ECT2 |  |  | 510 | 0.626 | 0.5258 | Yes |
| 184 | IQGAP3 |  |  | 516 | 0.621 | 0.5265 | Yes |
| 185 | GEN1 |  |  | 517 | 0.620 | 0.5286 | Yes |
| 186 | HAUS8 |  |  | 527 | 0.613 | 0.5282 | Yes |
| 187 | CDK2 |  |  | 530 | 0.612 | 0.5297 | Yes |
| 188 | ZGRF1 |  |  | 531 | 0.611 | 0.5317 | Yes |
| 189 | UPF3B |  |  | 536 | 0.610 | 0.5327 | Yes |
| 190 | BTG3 |  |  | 540 | 0.607 | 0.5339 | Yes |
| 191 | DDX39A |  |  | 542 | 0.605 | 0.5356 | Yes |
| 192 | RCC1 |  |  | 549 | 0.603 | 0.5360 | Yes |
| 193 | C18orf54 |  |  | 550 | 0.603 | 0.5380 | Yes |
| 194 | CENPQ |  |  | 569 | 0.590 | 0.5352 | Yes |
| 195 | MIS18BP1 |  |  | 574 | 0.588 | 0.5360 | Yes |
| 196 | CENPF |  |  | 575 | 0.588 | 0.5380 | Yes |
| 197 | RTTN |  |  | 577 | 0.588 | 0.5397 | Yes |
| 198 | CCNB1 |  |  | 579 | 0.587 | 0.5414 | Yes |
| 199 | TIMELESS |  |  | 591 | 0.583 | 0.5403 | Yes |
| 200 | CEP135 |  |  | 598 | 0.578 | 0.5406 | Yes |
| 201 | EXOSC8 |  |  | 604 | 0.576 | 0.5412 | Yes |
| 202 | SRPK1 |  |  | 610 | 0.574 | 0.5418 | Yes |
| 203 | SMC1A |  |  | 618 | 0.570 | 0.5418 | Yes |
| 204 | CKAP2 |  |  | 619 | 0.569 | 0.5437 | Yes |
| 205 | CDC25C |  |  | 626 | 0.568 | 0.5439 | Yes |
| 206 | ADCY3 |  |  | 630 | 0.566 | 0.5450 | Yes |
| 207 | HAT1 |  |  | 632 | 0.565 | 0.5466 | Yes |
| 208 | SPAG5 |  |  | 642 | 0.555 | 0.5460 | Yes |
| 209 | HAUS1 |  |  | 649 | 0.552 | 0.5463 | Yes |
| 210 | YEATS4 |  |  | 652 | 0.550 | 0.5475 | Yes |
| 211 | NUSAP1 |  |  | 654 | 0.550 | 0.5491 | Yes |
| 212 | SLC38A1 |  |  | 656 | 0.550 | 0.5507 | Yes |
| 213 | PAICS |  |  | 668 | 0.546 | 0.5495 | Yes |
| 214 | NPAT |  |  | 670 | 0.545 | 0.5511 | Yes |
| 215 | NUP107 |  |  | 675 | 0.539 | 0.5518 | Yes |
| 216 | TUBB |  |  | 678 | 0.538 | 0.5530 | Yes |
| 217 | WEE1 |  |  | 685 | 0.536 | 0.5532 | Yes |
| 218 | CIT |  |  | 690 | 0.535 | 0.5539 | Yes |
| 219 | NUP205 |  |  | 705 | 0.531 | 0.5519 | Yes |
| 220 | HADH |  |  | 706 | 0.530 | 0.5536 | Yes |
| 221 | DLGAP5 |  |  | 712 | 0.529 | 0.5541 | Yes |
| 222 | RIF1 |  |  | 713 | 0.529 | 0.5558 | Yes |
| 223 | CORO1C |  |  | 718 | 0.526 | 0.5565 | Yes |
| 224 | GPSM2 |  |  | 719 | 0.526 | 0.5582 | Yes |
| 225 | BAZ1B |  |  | 721 | 0.525 | 0.5597 | Yes |
| 226 | HAUS6 |  |  | 724 | 0.525 | 0.5609 | Yes |
| 227 | NASP |  |  | 741 | 0.518 | 0.5583 | No |
| 228 | LINC01224 |  |  | 760 | 0.513 | 0.5552 | No |
| 229 | POLQ |  |  | 771 | 0.508 | 0.5542 | No |
| 230 | NUP62 |  |  | 777 | 0.505 | 0.5545 | No |
| 231 | DCLRE1C |  |  | 786 | 0.503 | 0.5540 | No |
| 232 | CCDC150 |  |  | 804 | 0.496 | 0.5511 | No |
| 233 | AHI1 |  |  | 814 | 0.494 | 0.5503 | No |
| 234 | LIN54 |  |  | 823 | 0.490 | 0.5498 | No |
| 235 | SRSF10 |  |  | 831 | 0.489 | 0.5495 | No |
| 236 | ACYP1 |  |  | 834 | 0.489 | 0.5506 | No |
| 237 | CDC20 |  |  | 844 | 0.486 | 0.5498 | No |
| 238 | SMPD4 |  |  | 852 | 0.484 | 0.5495 | No |
| 239 | UBR7 |  |  | 857 | 0.483 | 0.5500 | No |
| 240 | RND3 |  |  | 860 | 0.482 | 0.5511 | No |
| 241 | TUBA1B |  |  | 862 | 0.481 | 0.5524 | No |
| 242 | ARL6IP6 |  |  | 880 | 0.476 | 0.5494 | No |
| 243 | DEPDC1B |  |  | 906 | 0.470 | 0.5443 | No |
| 244 | ZNF90 |  |  | 921 | 0.466 | 0.5420 | No |
| 245 | ITGB3BP |  |  | 935 | 0.462 | 0.5401 | No |
| 246 | HIRIP3 |  |  | 936 | 0.462 | 0.5416 | No |
| 247 | LIN9 |  |  | 949 | 0.459 | 0.5399 | No |
| 248 | BAZ1A |  |  | 960 | 0.455 | 0.5387 | No |
| 249 | KIF22 |  |  | 975 | 0.450 | 0.5364 | No |
| 250 | FANCD2 |  |  | 976 | 0.450 | 0.5379 | No |
| 251 | CASP8AP2 |  |  | 1025 | 0.436 | 0.5264 | No |
| 252 | ZNF519 |  |  | 1027 | 0.436 | 0.5276 | No |
| 253 | FANCL |  |  | 1052 | 0.427 | 0.5226 | No |
| 254 | CEP57 |  |  | 1055 | 0.426 | 0.5234 | No |
| 255 | DLEU2 |  |  | 1059 | 0.426 | 0.5240 | No |
| 256 | ARHGAP11B |  |  | 1069 | 0.424 | 0.5230 | No |
| 257 | JADE1 |  |  | 1089 | 0.419 | 0.5193 | No |
| 258 | NEK2 |  |  | 1100 | 0.417 | 0.5180 | No |
| 259 | KCTD9 |  |  | 1104 | 0.416 | 0.5186 | No |
| 260 | CTDSPL2 |  |  | 1109 | 0.415 | 0.5189 | No |
| 261 | TMEM245 |  |  | 1120 | 0.414 | 0.5176 | No |
| 262 | CBX5 |  |  | 1132 | 0.412 | 0.5160 | No |
| 263 | BUB1 |  |  | 1145 | 0.408 | 0.5141 | No |
| 264 | HNRNPD |  |  | 1177 | 0.401 | 0.5071 | No |
| 265 | DDB2 |  |  | 1189 | 0.399 | 0.5054 | No |
| 266 | SACS |  |  | 1195 | 0.397 | 0.5054 | No |
| 267 | SCLT1 |  |  | 1201 | 0.397 | 0.5054 | No |
| 268 | H1F0 |  |  | 1229 | 0.391 | 0.4994 | No |
| 269 | PTMA |  |  | 1233 | 0.390 | 0.4999 | No |
| 270 | APOLD1 |  |  | 1239 | 0.389 | 0.4998 | No |
| 271 | PSMC3 |  |  | 1241 | 0.389 | 0.5008 | No |
| 272 | STMN1 |  |  | 1249 | 0.387 | 0.5002 | No |
| 273 | HIST1H2BH |  |  | 1321 | 0.375 | 0.4823 | No |
| 274 | HMGXB4 |  |  | 1322 | 0.375 | 0.4836 | No |
| 275 | STK17B |  |  | 1324 | 0.374 | 0.4846 | No |
| 276 | ANP32E |  |  | 1334 | 0.371 | 0.4834 | No |
| 277 | RNASEH2A |  |  | 1341 | 0.369 | 0.4830 | No |
| 278 | TOPBP1 |  |  | 1355 | 0.366 | 0.4807 | No |
| 279 | RALY |  |  | 1368 | 0.362 | 0.4787 | No |
| 280 | EIF3D |  |  | 1405 | 0.357 | 0.4701 | No |
| 281 | SPATA5 |  |  | 1424 | 0.352 | 0.4665 | No |
| 282 | CNTROB |  |  | 1528 | 0.335 | 0.4398 | No |
| 283 | CLIC1 |  |  | 1552 | 0.331 | 0.4347 | No |
| 284 | CDC27 |  |  | 1562 | 0.330 | 0.4334 | No |
| 285 | FUS |  |  | 1571 | 0.329 | 0.4323 | No |
| 286 | TMEM237 |  |  | 1576 | 0.328 | 0.4323 | No |
| 287 | TPM4 |  |  | 1578 | 0.328 | 0.4331 | No |
| 288 | SESTD1 |  |  | 1613 | 0.321 | 0.4250 | No |
| 289 | UTRN |  |  | 1615 | 0.321 | 0.4258 | No |
| 290 | EFNA5 |  |  | 1624 | 0.320 | 0.4247 | No |
| 291 | DLEU1 |  |  | 1638 | 0.318 | 0.4223 | No |
| 292 | TMX1 |  |  | 1703 | 0.310 | 0.4061 | No |
| 293 | HMGB1 |  |  | 1720 | 0.308 | 0.4028 | No |
| 294 | BAHCC1 |  |  | 1728 | 0.307 | 0.4019 | No |
| 295 | ENOSF1 |  |  | 1823 | 0.296 | 0.3775 | No |
| 296 | CNTRL |  |  | 1830 | 0.294 | 0.3769 | No |
| 297 | ILF2 |  |  | 1833 | 0.294 | 0.3773 | No |
| 298 | SRSF3 |  |  | 1834 | 0.294 | 0.3783 | No |
| 299 | STK39 |  |  | 1871 | 0.289 | 0.3696 | No |
| 300 | ODF2 |  |  | 1887 | 0.287 | 0.3665 | No |
| 301 | LIN52 |  |  | 1921 | 0.283 | 0.3585 | No |
| 302 | COPS8 |  |  | 1951 | 0.279 | 0.3516 | No |
| 303 | E2F3 |  |  | 1961 | 0.277 | 0.3501 | No |
| 304 | PIP4K2A |  |  | 1981 | 0.275 | 0.3459 | No |
| 305 | TNPO3 |  |  | 1989 | 0.274 | 0.3449 | No |
| 306 | KITLG |  |  | 2019 | 0.270 | 0.3380 | No |
| 307 | SRBD1 |  |  | 2028 | 0.269 | 0.3368 | No |
| 308 | SKA2 |  |  | 2061 | 0.266 | 0.3290 | No |
| 309 | MPHOSPH9 |  |  | 2082 | 0.264 | 0.3245 | No |
| 310 | CEP192 |  |  | 2109 | 0.262 | 0.3184 | No |
| 311 | CKAP5 |  |  | 2135 | 0.258 | 0.3125 | No |
| 312 | PIGK |  |  | 2159 | 0.256 | 0.3071 | No |
| 313 | GPC6 |  |  | 2232 | -0.251 | 0.2885 | No |
| 314 | PTPRE |  |  | 2265 | -0.255 | 0.2808 | No |
| 315 | KIZ |  |  | 2301 | -0.259 | 0.2722 | No |
| 316 | EFS |  |  | 2326 | -0.262 | 0.2666 | No |
| 317 | USP33 |  |  | 2422 | -0.273 | 0.2419 | No |
| 318 | ILDR2 |  |  | 2478 | -0.280 | 0.2280 | No |
| 319 | PCNP |  |  | 2585 | -0.299 | 0.2004 | No |
| 320 | PCM1 |  |  | 2715 | -0.320 | 0.1666 | No |
| 321 | ZDHHC17 |  |  | 2718 | -0.320 | 0.1672 | No |
| 322 | GAS2L3 |  |  | 2755 | -0.327 | 0.1586 | No |
| 323 | NGLY1 |  |  | 2766 | -0.330 | 0.1570 | No |
| 324 | HERPUD1 |  |  | 2777 | -0.331 | 0.1554 | No |
| 325 | PIP5K1B |  |  | 2849 | -0.344 | 0.1374 | No |
| 326 | ZNF43 |  |  | 2872 | -0.348 | 0.1326 | No |
| 327 | FIGN |  |  | 2879 | -0.351 | 0.1321 | No |
| 328 | SMARCA2 |  |  | 2903 | -0.356 | 0.1271 | No |
| 329 | PCDH17 |  |  | 2924 | -0.360 | 0.1229 | No |
| 330 | RSRC1 |  |  | 2966 | -0.367 | 0.1131 | No |
| 331 | MXI1 |  |  | 3000 | -0.372 | 0.1054 | No |
| 332 | APBB2 |  |  | 3023 | -0.378 | 0.1007 | No |
| 333 | ABAT |  |  | 3075 | -0.391 | 0.0883 | No |
| 334 | TMSB4X |  |  | 3141 | -0.412 | 0.0721 | No |
| 335 | PIK3R3 |  |  | 3151 | -0.414 | 0.0711 | No |
| 336 | HMGN5 |  |  | 3173 | -0.421 | 0.0668 | No |
| 337 | PDZRN3 |  |  | 3276 | -0.449 | 0.0408 | No |
| 338 | BEST3 |  |  | 3302 | -0.458 | 0.0356 | No |
| 339 | ST8SIA4 |  |  | 3406 | -0.500 | 0.0095 | No |
| 340 | ARX |  |  | 3443 | -0.516 | 0.0015 | No |
| 341 | GLCCI1 |  |  | 3444 | -0.517 | 0.0032 | No |
| 342 | ARL4D |  |  | 3453 | -0.521 | 0.0028 | No |
| 343 | CDK6 |  |  | 3466 | -0.526 | 0.0013 | No |
| 344 | H3F3A |  |  | 3471 | -0.528 | 0.0020 | No |
| 345 | LRP6 |  |  | 3473 | -0.530 | 0.0035 | No |
| 346 | ASAP1 |  |  | 3484 | -0.534 | 0.0025 | No |
| 347 | KLHL13 |  |  | 3486 | -0.534 | 0.0040 | No |
| 348 | ATF7IP |  |  | 3525 | -0.555 | -0.0044 | No |
| 349 | AASS |  |  | 3539 | -0.564 | -0.0060 | No |
| 350 | ARL6IP1 |  |  | 3574 | -0.586 | -0.0132 | No |
| 351 | FBXW7 |  |  | 3629 | -0.626 | -0.0257 | No |
| 352 | CDCA7L |  |  | 3642 | -0.637 | -0.0268 | No |
| 353 | MEIS1 |  |  | 3651 | -0.648 | -0.0268 | No |
| 354 | CHD6 |  |  | 3652 | -0.648 | -0.0246 | No |
| 355 | BCL11B |  |  | 3656 | -0.651 | -0.0233 | No |
| 356 | GAD1 |  |  | 3704 | -0.690 | -0.0337 | No |
| 357 | MDK |  |  | 3706 | -0.691 | -0.0316 | No |
| 358 | RGS12 |  |  | 3714 | -0.697 | -0.0312 | No |
| 359 | LCORL |  |  | 3718 | -0.698 | -0.0297 | No |
| 360 | FAT1 |  |  | 3730 | -0.708 | -0.0303 | No |
| 361 | DLX6 |  |  | 3735 | -0.711 | -0.0290 | No |
| 362 | NRXN3 |  |  | 3738 | -0.714 | -0.0272 | No |
| 363 | ZNF704 |  |  | 3750 | -0.729 | -0.0277 | No |
| 364 | TOP2B |  |  | 3777 | -0.767 | -0.0322 | No |
| 365 | SOX4 |  |  | 3797 | -0.786 | -0.0347 | No |
| 366 | SPECC1 |  |  | 3801 | -0.790 | -0.0329 | No |
| 367 | MSI2 |  |  | 3816 | -0.802 | -0.0340 | No |
| 368 | MAGI1 |  |  | 3827 | -0.815 | -0.0340 | No |
| 369 | PFN2 |  |  | 3841 | -0.832 | -0.0347 | No |
| 370 | CCDC88A |  |  | 3854 | -0.848 | -0.0351 | No |
| 371 | ZEB2 |  |  | 3876 | -0.894 | -0.0378 | No |
| 372 | CHD7 |  |  | 3880 | -0.904 | -0.0356 | No |
| 373 | PROM1 |  |  | 3892 | -0.929 | -0.0355 | No |
| 374 | DSEL |  |  | 3897 | -0.940 | -0.0334 | No |
| 375 | SP9 |  |  | 3898 | -0.942 | -0.0303 | No |
| 376 | FBLN1 |  |  | 3909 | -0.965 | -0.0298 | No |
| 377 | RAB3IP |  |  | 3940 | -1.037 | -0.0344 | No |
| 378 | DCLK2 |  |  | 3945 | -1.043 | -0.0320 | No |
| 379 | SOX2 |  |  | 3953 | -1.069 | -0.0304 | No |
| 380 | SOX6 |  |  | 3956 | -1.081 | -0.0273 | No |
| 381 | NEDD4L |  |  | 3960 | -1.090 | -0.0245 | No |
| 382 | NCAM1 |  |  | 3966 | -1.106 | -0.0222 | No |
| 383 | CDH2 |  |  | 3998 | -1.233 | -0.0264 | No |
| 384 | ZBTB20 |  |  | 4024 | -1.407 | -0.0285 | No |
| 385 | LSAMP |  |  | 4028 | -1.442 | -0.0245 | No |
| 386 | DLX1 |  |  | 4041 | -1.558 | -0.0225 | No |
| 387 | DLX2 |  |  | 4055 | -1.704 | -0.0204 | No |
| 388 | INSM1 |  |  | 4059 | -1.733 | -0.0154 | No |
| 389 | ASCL1 |  |  | 4061 | -1.762 | -0.0098 | No |
| 390 | DLX5 |  |  | 4090 | -2.619 | -0.0087 | No |
| 391 | LMO1 |  |  | 4093 | -3.174 | 0.0013 | No |
Table: GSEA details [plain text format]

  

Fig 2: RICHARDS\_NOWAKOWSKI\_SCIENCE\_2017\_MGE\_IPC2\_UPREG: Random ES distribution      
 Gene set null distribution of ES for **RICHARDS\_NOWAKOWSKI\_SCIENCE\_2017\_MGE\_IPC2\_UPREG**
